# Adult intestinal malrotation presenting as caecal volvulus with incidental findings of duplicate inferior vena cava and other rare abnormalities: case report

**DOI:** 10.1093/jscr/rjae132

**Published:** 2024-03-08

**Authors:** Samuel R Thomson, Sam M Hanna, Amit Sarkar, Atandrila Das, Dayashan S Perera

**Affiliations:** Department of Surgery, St George Hospital, Gray St, Kogarah, Sydney, NSW 2217, Australia; Department of Surgery, St George Hospital, Gray St, Kogarah, Sydney, NSW 2217, Australia; Department of Surgery, St George Hospital, Gray St, Kogarah, Sydney, NSW 2217, Australia; Department of Surgery, St George Hospital, Gray St, Kogarah, Sydney, NSW 2217, Australia; Department of Surgery, St George Hospital, Gray St, Kogarah, Sydney, NSW 2217, Australia

**Keywords:** caecal volvulus, intestinal malrotation, duplicate inferior vena cava, polysplenia syndrome

## Abstract

Intestinal malrotation and duplication of the inferior vena cava are rarely diagnosed in adult patients; however, incidence is likely underestimated as they are usually asymptomatic. These congenital malformations have been previously reported in the same patient twice but never with colonic obstruction or ischaemia. A 25-year-old female presented with nausea, vomiting, obstipation, and abdominal pain, and on computed tomography of the abdomen and pelvis was diagnosed with a caecal volvulus and pneumatosis coli associated with intestinal malrotation requiring emergency right hemicolectomy. Incidentally, the patient was noted to have duplication of the inferior vena cava, azygos continuation of the inferior vena cava, and splenic fragmentation. This constellation of symptoms has not been reported in the literature previously. The pattern of malformations follows that of polysplenia syndrome. Although rare, awareness of these malformations can be useful to clinicians.

## Introduction

Intestinal malrotation usually presents in early childhood, 85% of cases within the first 2 weeks of life [1]. Incidence in adults is likely underestimated as most are asymptomatic, some present chronically with vague abdominal pain and a minority present acutely due to obstruction or ischemia. Similarly, the incidence of duplicate inferior vena cava (DIVC) is also likely underestimated as this congenital anomaly is mostly diagnosed incidentally, reported incidence ranging from 0.2% to 3% [2]. These congenital abnormalities have been previously reported in the same patient but without obstruction or ischemia [3, 4]. Here, we present an adult with an acute caecal volvulus associated with intestinal malrotation as well as other incidentally noted congenital abnormalities, including DIVC, azygos continuation of the IVC, and splenic fragmentation.

### Case report

A 25-year-old female presented to the emergency department with 2 days of worsening nausea, vomiting, and abdominal pain. The initial symptoms were postprandial nausea and generalised abdominal pain, which progressed to episodes of vomiting, obstipation, and localised left upper quadrant abdominal pain. There was no other significant medical or surgical history. The vital signs were within normal limits, the patient’s abdomen was soft with tenderness in the left upper quadrant and left flank.

Blood test results were largely unremarkable, the haemoglobin was 128 g/l, WCC was 6.1 × 10^9^/l and C-reactive protein was 2 mg/l. The venous lactate was minimally elevated at 2.4 mmol/l.

**Figure 1:**
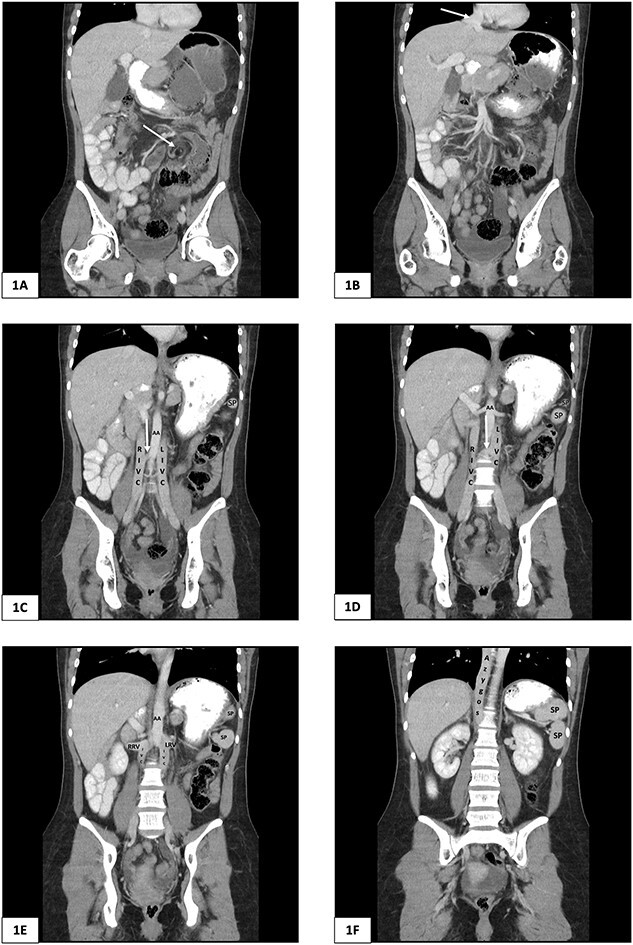
Computed tomography of the abdomen and pelvis of 25-year-old female presenting with caecal volvulus, intestinal malrotation, duplicate inferior vena cava, azygos continuation of the IVC, and fragmentation of the spleen. RIVC, right inferior vena cava; LIVC, left inferior vena cava; RRV, right renal vein; LRV, left renal vein; AA, abdominal aorta; SP, splenic fragment. (A) Arrow indicates mesenteric whirl sign. (B) Arrow indicates suprahepatic IVC draining from right, middle, and left hepatic veins into the right atrium. (C) and (D) Arrow indicates communicating vein from right IVC to left IVC.

**Figure 2:**
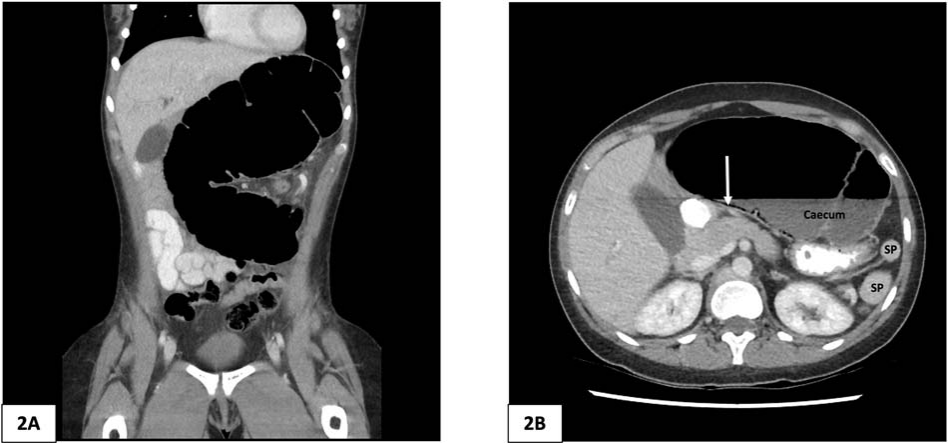
Computed tomography of the abdomen and pelvis of 25-year-old female patient. (A) Coronal slice demonstrating caecal volvulus with distended loop of colon. (B) Axial slice demonstrating 97 mm diameter distended caecum, multiple splenic fragments, arrow indicates pneumatosis coli, indicating colonic ischaemia. SP, spleen fragments.

A plain abdominal X-ray was performed showing a grossly distended loop of colon in the left upper abdomen. Subsequent computed tomography (CT) scan of the abdomen and pelvis was obtained using oral and intravenous contrast with images acquired during portal venous phase. The CT showed a mesenteric whirl sign (Fig. 1A) and caecal volvulus with the caecum measuring 97 mm diameter at its most distended point with associated pneumatosis coli (Fig. 2A and B).

There was malrotation of the gut with all colon on the left side and most of the small bowel on the right side of the abdomen (Fig. 2A). There was duplication of the infrarenal IVC, interruption of the suprarenal IVC with azygos continuation (Fig. 1C–F). A left-sided IVC draining venous blood from the left common iliac vein was present which drained into the left renal vein, while the right IVC drained blood from the right common iliac vein into the right renal vein. A retroaortic communication draining blood from the right IVC into the left IVC was also noted. From the renal veins all venous return was via the azygos vein to the superior vena cava with interruption of the suprarenal IVC. A suprahepatic IVC draining blood from the middle, left, and right hepatic veins into the right atrium was present (Fig. 1B). The spleen was divided into five distinct fragments, all within the splenic fossa and each supplied by an individual branch of the splenic artery (Figs 1 and 2).

The patient proceeded to undergo an emergency laparotomy. The intraoperative findings were a malrotated midgut with small bowel on the right side of the abdomen and colon on the left, there was a type 2 caecal volvulus with torsion of the caecum and part of the terminal ileum. The ascending colon was completely mobile without any attachment to the retroperitoneal structures. The serosa of the torted caecum had split over the caecal pole and an open right hemicolectomy and stapled ileocolic anastomosis was performed. The patients postoperative recovery was unremarkable and they were discharged 4 days post surgery.

## Discussion

We present a case of a 25-year-old female with an acute abdomen due to a caecal volvulus requiring emergency right hemicolectomy with multiple abnormalities. Congenital malformations noted in this case were intestinal malrotation, duplication of the IVC, azygous continuation of the IVC, and splenic fragmentation. To our knowledge, there have been no previously reported cases in the literature with this constellation of abnormal findings and none resulting in an acute presentation requiring emergency surgery.

Intestinal malrotation occurs due to failure of the midgut to undergo a 270° counter clockwise rotation around the superior mesenteric artery during embryonic development and can result in a narrow-based mesentery and decreased attachment of the caecum and ascending colon posteriorly [5]. Our case was noted to have a mobile caecum and ascending colon. Caecal volvulus associated with midgut malrotation is rare, especially in adults, but has been reported in various case reports [6–9].

Variations in the anatomy of the IVC occur due to abnormal regression or persistence of primitive veins in the developing embryo, usually between weeks 4–8 of gestation [10]. Duplication of the IVC occurs when regression of the left supracardinal vein does not occur during organogenesis [11]. Two cases of simultaneous DIVC and intestinal malrotation have been reported in the literature previously, although neither was associated with caecal volvulus or intestinal obstruction [3, 4]. In our case the left and right IVC were of equal diameter with a communication draining from the right IVC to the left IVC, a type 2c DIVC as described by Morita *et al.* [12].

The described abnormalities of our case follow the pattern of polysplenia syndrome which is defined as two or more spleens accompanying congenital malformations in the abdomen or thorax that arise between weeks 4–6 of embryogenesis [13, 14]. IVC abnormalities and intestinal malrotation are known abnormalities associated with polysplenia syndrome [14]. However, polysplenia is rare in adulthood as most cases also have severe congenital cardiac malformations, which result in 75% mortality by 5 years old [15]. Our case had no known cardiac malformations and had not required cardiac interventions as a child. Interestingly the two other case reports to describe concurrent DIVC and intestinal malrotation did not have polysplenia, but each case had at least one other congenital abnormality.

Although rare, awareness of various congenital malformations and their associations can be useful in interpreting cross sectional imaging and planning for surgical and interventional procedures. With further reporting of cases our understanding of these syndromes and associations will continue to improve.
